# Chemotherapy-Response Monitoring of Breast Cancer Patients Using Quantitative Ultrasound-Based Intra-Tumour Heterogeneities

**DOI:** 10.1038/s41598-017-09678-0

**Published:** 2017-09-04

**Authors:** Ali Sadeghi-Naini, Lakshmanan Sannachi, Hadi Tadayyon, William T. Tran, Elzbieta Slodkowska, Maureen Trudeau, Sonal Gandhi, Kathleen Pritchard, Michael C. Kolios, Gregory J. Czarnota

**Affiliations:** 1 Department of Medical Biophysics, University of Toronto, Toronto, ON Canada; 2 Physical Sciences, Sunnybrook Research Institute, Sunnybrook Health Sciences Centre, Toronto, ON Canada; 3 Department of Radiation Oncology, Odette Cancer Centre, Sunnybrook Health Sciences Centre, Toronto, ON Canada; 4 Department of Radiation Oncology, University of Toronto, Toronto, ON Canada; 5 Centre for Health and Social Care Research, Sheffield Hallam University, Sheffield, UK; 6 Division of Anatomic Pathology, Sunnybrook Health Sciences Centre, Toronto, ON Canada; 7 Division of Medical Oncology, Sunnybrook Health Sciences Centre, Toronto, ON Canada; 8 Department of Physics, Ryerson University, Toronto, ON Canada

## Abstract

Anti-cancer therapies including chemotherapy aim to induce tumour cell death. Cell death introduces alterations in cell morphology and tissue micro-structures that cause measurable changes in tissue echogenicity. This study investigated the effectiveness of quantitative ultrasound (QUS) parametric imaging to characterize intra-tumour heterogeneity and monitor the pathological response of breast cancer to chemotherapy in a large cohort of patients (n = 100). Results demonstrated that QUS imaging can non-invasively monitor pathological response and outcome of breast cancer patients to chemotherapy early following treatment initiation. Specifically, QUS biomarkers quantifying spatial heterogeneities in size, concentration and spacing of acoustic scatterers could predict treatment responses of patients with cross-validated accuracies of 82 ± 0.7%, 86 ± 0.7% and 85 ± 0.9% and areas under the receiver operating characteristic (ROC) curve of 0.75 ± 0.1, 0.80 ± 0.1 and 0.89 ± 0.1 at 1, 4 and 8 weeks after the start of treatment, respectively. The patients classified as responders and non-responders using QUS biomarkers demonstrated significantly different survivals, in good agreement with clinical and pathological endpoints. The results form a basis for using early predictive information on survival-linked patient response to facilitate adapting standard anti-cancer treatments on an individual patient basis.

## Introduction

Breast cancer is the most common malignancy among women in developed countries^1^. Up to 20% of newly-diagnosed breast cancer patients each year present with locally advanced breast cancer (LABC)^2–4^. LABC includes stage III and a subset of stage IIB disease and often presents as tumours greater than 5 cm in size that may involve the skin and/or chest wall or be classified as inflammatory breast cancer. Women with locally-advanced breast cancer have poor long term survival rates compared to early stage breast cancer patients^5^. The standard treatment for these patients includes neoadjuvant chemotherapy to reduce the size of the tumour, followed by surgery and, if required, radiation and/or hormonal therapy^6–8^. Current methods for monitoring response to neoadjuvant chemotherapy rely on gross changes in tumor size based on physical examination or conventional anatomical imaging including X-ray mammography and T1/T2-wighted magnetic resonance imaging (MRI). A major limitation associated with these methods is that changes in tumor size frequently require up to months of therapy and in some cases a mass diminishment is not present despite a positive pathological response to neoadjuvant chemotherapy^9^. Neither are used routinely to monitor response to neoadjuvant chemotherapy in a clinical setting. The standard approach to determine tumour pathological response to neoadjuvant chemotherapy is based on post-surgical histopathology. Systematic techniques such as the Miller and Payne (MP) score are used to describe pathological response to chemotherapy based on the cellular and histopathological characteristics of tumour. The MP score quantifies changes in tumour cellularity in response to therapy based on pre-treatment core biopsy and post-treatment surgical specimens^10^. Pathological response of tumour to neoadjuvant chemotherapy has been demonstrated to be highly correlated with improved overall survival^7, 11–15^. However, previous studies on post-surgical evaluation of LABC response to neoadjuvant chemotherapy reported that over 40% of patients may demonstrate poor pathological response to treatment^16^. This highlights the importance of early predictions of patient response to chemotherapy, since this can facilitate an opportunity to modify ineffective treatments to more effective ones on an individual patient basis, before it is potentially too late^17–20^.

Functional imaging techniques that can detect changes in tumour micro-structure and physiology in response to treatment could provide early assessments of therapy response^21–24^. In this context, a number of functional imaging modalities, including positron emission tomography (PET) and MRI, have recently been demonstrated capable of evaluating cancer treatment response within weeks after treatment initiation^25–28^. However, these modalities are often costly with long scan times and require administration of exogenous contrast agents to detect response-related changes in tumour. Such factors potentially limit the availability and applicability of these modalities for treatment response monitoring as well as the number of times that a patient can be scanned during a course of treatment. On the other hand, ultrasound is a relatively inexpensive and rapid imaging modality that relies on the micro-structural and physical properties of tissue to generate contrast and, therefore, does not require any exogenous contrast agents to measure changes in cells and tissue microstructure in response to treatment^29–31^.

Quantitative ultrasound (QUS) techniques have previously been used in several tissue characterization applications. In particular, QUS parameters derived based on the spectral analysis of phase-resolved ultrasound radio-frequency (RF) signal have been applied in the diagnosis of ocular and prostate cancers, the examination of liver and renal tissues, studies of cardiac and vascular abnormalities, and the characterization of breast masses^32–35^. Measures of ultrasound signal attenuation have also been used to classify different tissue types and to detect diseases and abnormalities. Particularly, the attenuation coefficient estimate (ACE) parameter has been used to differentiate benign lesions from normal breast tissue, and to classify different tissue types in the breast such as fat, parenchyma and invasive ductal carcinoma^36, 37^. In addition, measures of acoustic scatterer spacing have been used to characterize tissues with detectable periodicity in their structural organization. Specifically, the spacing among scatterers (SAS) parameter has been applied to characterize diffuse disease of the liver^38–40^, to differentiate normal and pathological breast tissues, and to classify breast tumours in terms of histological grade^41, 42^. In a number of preclinical studies, QUS spectral analysis techniques at low (3–9 MHz) and high (15–50 MHz) frequencies have been demonstrated to be effective in detecting tumour response to a variety of anti-cancer therapies including chemotherapy, photodynamic therapy, radiation therapy and ultrasound-stimulated microbubble therapy^43–50^. In recent pilot clinical studies, QUS techniques have been applied to evaluate breast cancer response to neoadjuvant chemotherapy within weeks after the start of treatment and demonstrated good correlations with clinical response identified at the end of treatment^51, 52^. In those studies, changes in the QUS parameters, including the mid-band fit (MBF), slope, intercept, average scatter diameter (ASD), and average acoustic concentration (AAC), were related to alterations in cell ensembles and tissue microstructure in response to treatment. Most recently, in addition to QUS mean-value parameters, textural features extracted from QUS parametric maps have been investigated for tumour response monitoring in animal tumour models and small patient populations^53, 54^. These textural parameters, which quantify the spatial relationship between local acoustic properties within tissue microstructures, have been demonstrated to be capable of characterizing intra-tumour response heterogeneities^55^. Combined mean-value and textural parameters of the QUS parametric maps improved accuracies for detecting tumour response to treatment in a small pilot study^54^.

The aim of this study was to develop and evaluate multi-parametric QUS models to predict pathological response of LABC tumours after the start of neoadjuvant chemotherapy. The sample size here was expanded to a large cohort of 100 LABC patients and QUS parameters such as MBF, slope, intercept, ACE, SAS, ASD, and AAC, as well as their corresponding textural features such as contrast, correlation, homogeneity, and energy were investigated using volumetric ultrasound scans of patients. Multi-parametric QUS models for response evaluation was developed using k-nearest neighbours (KNN) classifiers in conjunction with a sequential forward feature selection method and a leave-one-patient-out scheme for cross-validation.

## Material and Methods

### Study Protocol

This study was conducted in accordance with institutional research ethics approval from Sunnybrook Health Sciences Centre, Toronto, ON, Canada. The study was open to all women with LABC aged 18–85 who were planned for neoadjuvant chemotherapy followed by a mastectomy or lumpectomy. Patients who failed to attend two or more of the ultrasound scans (described below) were eliminated from the study. In keeping with this, 100 eligible patients were recruited for the study after obtaining written informed consent and completed their participation in the study. All patients underwent a core needle biopsy to confirm cancer diagnosis (based on radiology reports), and to determine histological subtype, cellularity and hormone receptor status of the tumour. Pre-treatment magnetic resonance (MR) images of the breast were acquired for each patient to determine initial tumour size. Ultrasound scans were performed immediately before the start of chemotherapy (after pre-treatment MRI) and at weeks 1, 4, and 8 after the start of treatment. The scans were performed by three experienced sonographers following a standardized protocol for data acquisition (described below). All scans for each patient were typically done by the same sonographer. Ultrasound data was acquired with patients lying supine with their arms above their heads. The sonographers were blinded to patient tumour-response characteristics and study results. Patients were also followed clinically up to 5 years after their treatment and their clinical data were recorded for recurrence-free survival analysis.

### Pathological Response Evaluation

All patients underwent breast surgery after the completion of neoadjuvant chemotherapy, based on institutional guidelines. The majority of patients (~70%) underwent a mastectomy and about 30% of the patients went through a lumpectomy (breast conserving surgery). The average time between the first ultrasound scan (baseline) and surgery was about 22 weeks. Following surgery, the surgical specimens were prepared on whole-mount 5″ × 7″ pathology slides and stained with hematoxylin and eosin (H&E). The slides were digitized using a confocal scanner (TISSUEscope^TM^, Huron Technologies, Waterloo, ON). All pathology samples were examined by a board-certified pathologist who remained blinded to the study results. Patients were classified into two groups based on the identified Miller-Payne scores from pathology reports; responders (R): patients with Miller-Payne scores of 3–5 with more than 30% reduction in tumour cellularity in response to chemotherapy, and non-responders (NR): patients with Miller-Payne scores of 1 or 2, who demonstrated minimal changes in tumour cellularity (less than 30%) after chemotherapy^10^.

### Ultrasound Data Acquisition

Ultrasound data were acquired with a RF-enabled Sonix RP clinical research system (Ultrasonix, Vancouver, Canada) equipped with a linear array transducer L14–5/60, operating at a centre frequency of ~6 MHz with a −6 dB bandwidth range of 3–8 MHz. The RF data were collected with a 40 MHz sampling frequency and digitized with 16-bit resolution. The sizes of each image frame were 6 cm and 4–6 cm along lateral and axial directions, respectively, storing 512 RF lines laterally. For each breast tumour, four to seven image planes were typically acquired at approximately 1 cm intervals across the involved breast, with the focal depth set at the centre of the tumour. Although the majority of tumours were readily visible with ultrasound, the tumour location was also verified using the MR images.

### Ultrasound Data Analysis

Analysis of Ultrasound RF data was performed offline using quantitative ultrasound spectral analysis to derive mid-band fit (MBF), slope, intercept, spacing among scatterers (SAS), average scatterer diameter (ASD), and average acoustic concentration (AAC) parameters^41, 48, 51, 56^. The attenuation coefficient estimate (ACE) of tumour was calculated using a spectral difference method by estimating the rate of change in the spectral power magnitude with depth (over the tumour region) and frequency relative to a reference phantom with a known attenuation coefficient^57^. A reference phantom technique was used to normalize effects of the system transfer function, transducer beam-forming, and diffraction artefacts^56, 58^. The reference phantom was composed of 5 to 30 μm diameter glass beads embedded in a homogeneous background of microscopic oil droplets in gelatin (Medical Physics Department, University of Wisconsin, USA). The attenuation coefficient and speed of sound parameters of the reference phantom were 0.576 dB/MHz.cm and 1488 m/s, respectively. In estimating spectral (MBF, slope, intercept) and backscatter (ASD and AAC) parameters, the ACE of tumour was used for attenuation correction of the power spectrum using the point attenuation compensation method^59^. A two-layer (intervening tissue and tumor) attenuation correction was performed using total attenuation estimation^57^. An attenuation coefficient of 1 dB/MHz.cm was assumed for intervening breast tissue based on ultrasound tomography measurements of the breast^60^. Spectral parameters were estimated using linear regression analysis on normalized power spectrum over −6 dB bandwidth of the transducer^61^. The backscatter parameters were derived from the backscatter coefficient using a spherical Gaussian form factor model^35, 62^. The SAS parameter was derived by computing the autocorrelation of the power spectrum estimated by an autoregressive (AR) model using Burg’s algorithm^40^. To estimate the SAS parameter from the peak position, a mean speed of sound of 1540 m/s was used for the entire breast tissue based on data reported in the literature^60^.

The spectral, backscatter and SAS parameters were extracted using a sliding window analysis approach (2 mm × 2 mm windows; 94% adjacent overlap in both axial and lateral directions) to generate parametric images^53, 54^. Ultrasound data were analyzed across all acquired imaging planes through the scan volume that included identifiable tumour regions. Analysis parameters were reported from data within a region of interest (ROI) covering the entire tumour area within the plane. Parameter values acquired from different imaging planes in each scan were reported as tumour volumetric averages.

### Texture Analysis

In addition to mean-values of QUS parametric maps, second-order textural features were extracted from the parametric images using a grey-level co-occurrence matrix (GLCM) method. The GLCM represents statistically the angular relationship between neighboring pixels as well as the distance between them^55^. Using the statistics provided by the GLCM for each image, four textural features including contrast, correlation, homogeneity, and energy were derived from the MBF, SS, SI, SAS, ASD and AAC parametric maps^54^. Concisely, the contrast parameter represents local gray-level variation of an image, the correlation parameter measures the linear dependency among neighbouring pixels, the energy parameter quantifies textural uniformity within neighbouring pixels, and the homogeneity parameter measures the incidence of pixel pairs of different intensities.

### Classification and Statistical Analysis

Changes in QUS parameters for each patient during the course of treatment were calculated using the corresponding parameters acquired at pre-treatment as the baseline. A K-nearest neighbour (KNN) classification was used to evaluate the efficacy of QUS parameters to differentiate pathological response of patients at different times after the start of treatment^63^. Cross-validated sensitivity, specificity and accuracy were calculated, in addition to the area under the receiver operating characteristic (ROC) curve, to measure the performance of the classification. Before performing classification analysis, the patients were randomly sub-sampled into 50 subsets with equal numbers of pathological responders and non-responders (n = 2 × 19) to compensate for the imbalance in the number of patients in the response populations (81 responders versus and 19 non-responders)^64^. The optimal multivariate QUS feature set for each scan time was determined using a sequential forward feature selection approach. The average cross-validated accuracy of response classification over the 50 subsets was used as the selection criteria. A leave-one-patient-out method was applied for cross validation of classification in each subset.

Survival analysis was performed to generate recurrence-free Kaplan-Meier survival curves for the two response populations. The patient response was determined at each imaging time during treatment (weeks 1, 4, and 8 of chemotherapy) based on the corresponding optimal QUS feature set, and at post-treatment based on histopathology. Survival curves were compared using a log-rank test to assess for statistically significant differences between the treatment outcomes.

## Results

Table 1 summarizes the clinical characteristics of patients and their histopathological response to chemotherapy. The patients had an average age of 49 years (SD = 11, range: 29–83 years), and an average tumour size of 5.9 cm (SD = 2.8, range: 2–14 cm) in the longest dimension. Histological tumour types were primarily invasive ductal carcinoma (95%) with 5% of cases diagnosed with invasive lobular carcinoma. Also, 63% of patients had tumours with positive estrogen and/or progesterone receptors (ER/PR+), 32% of patients had tumours with over-expression of Her-2-Neu receptor (HER2+), and 28% of patients had tumours with triple negative receptors. The majority of patients received a combined anthracycline and taxane-based chemotherapy (91%). Patients with ER+ tumours received hormonal therapy (tamoxifen) or an aromatase inhibitor (letrozole) in an adjuvant setting. No treatment regimen was modified based on the imaging findings in this observational study. Based on histopathology of surgical specimens at post-treatment, 19 patients had a Miller-Payne score of 1 or 2 and were classified as pathological non-responders (progressive or stable disease with less than 30% reduction in tumour cellularity), whereas 81 patients had a score of 3–5 and were categorized as pathological responders (complete pathological response or partial response with more than 30% reduction in tumour cellularity). No significant differences were observed in tumor size changes assessed through physical examination at weeks one, four, and eight of treatment between the responding and non-responding patients.

**Table 1:** Summary of patient characteristics and histopathological response to chemotherapy.

	Mean ± Standard Deviation/Percentage
**Age**	49 ± 11 years
**Initial Tumour Size**	5.9 ± 2.8 cm
**Histology**	
	Invasive Ductal Carcinoma: 95%
	Invasive Lobular Carcinoma: 5%
**Tumour Grade**	
	Grade I: 10%
	Grade II: 63%
	Grade III: 27%
**Molecular Features**	
	ER/PR+: 63%
	ER/PR+ & HER2−: 40%
	HER2+: 32%
	ER− & PR− & HER2+: 9%
	ER− & PR− & HER2−: 28%
**Residual Tumour Size**	3.1 ± 4.0 cm
**Miller-Payne Score**	
	MP 1: 7%
	MP 2: 12%
	MP 3: 41%
	MP 4: 15%
	MP 5: 23%
**Response**	
	Responder: 81%
	Non-responder: 19%

**Figure 1:**
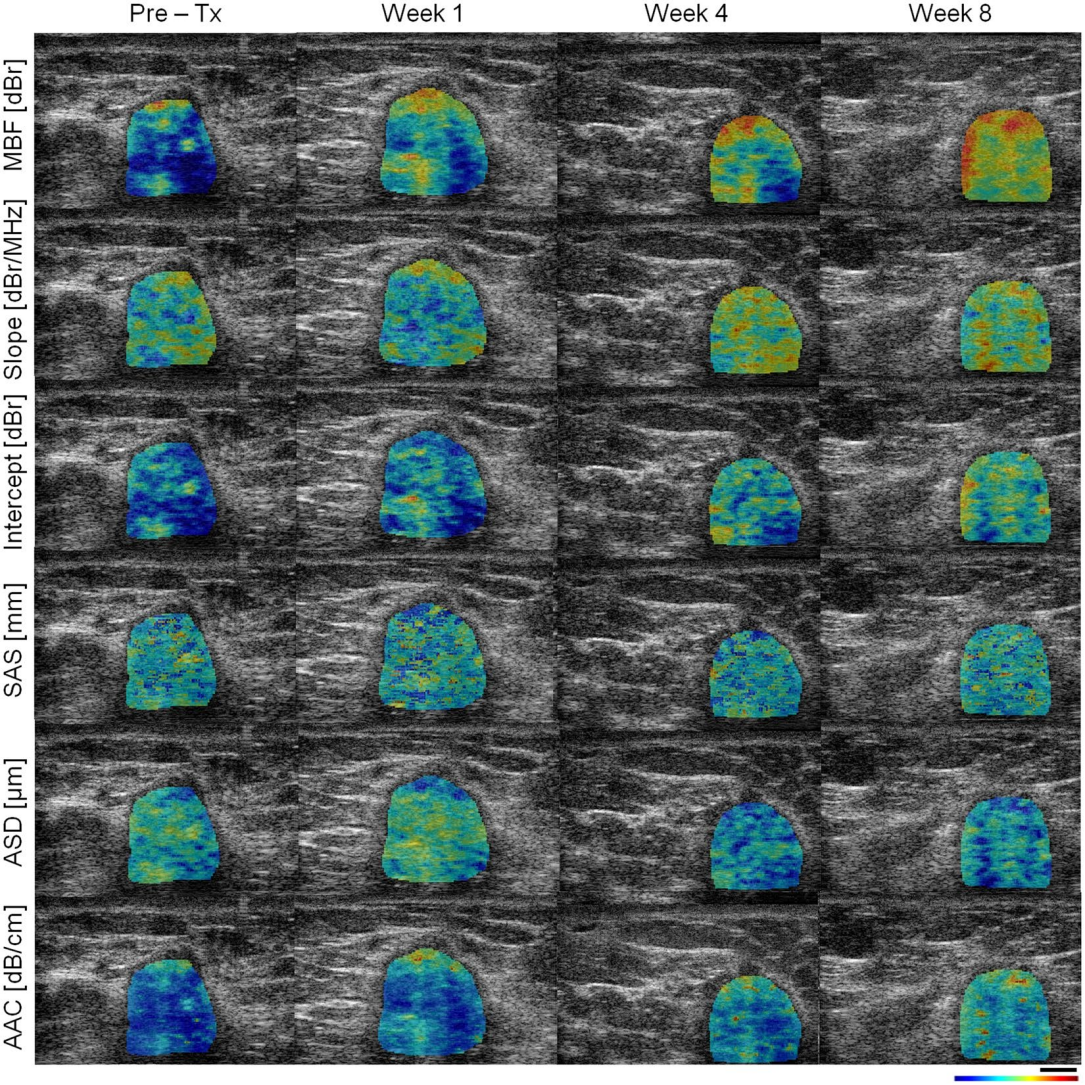
Representative ultrasound B-mode images with QUS parametric overlays acquired from a responding patient prior to and at different times after the start of chemotherapy. The parametric maps demonstrate spatial variations in quantitative parameters (MBF, slope, intercept, SAS, ASD, and AAC) that are linked to tumour micro-structure. Changes in mean-value and textural features of these maps were monitored over the course of treatment to evaluate patient response to chemotherapy. The color bar represents a scale encompassing 35 dBr for MBF, 10 dBr/MHz for slope, 65 dBr for intercept, 3 mm for SAS, 150 µm for ASD, and 55 dB/cm^3^ for AAC. The scale bar represents 5 mm.

**Figure 2:**
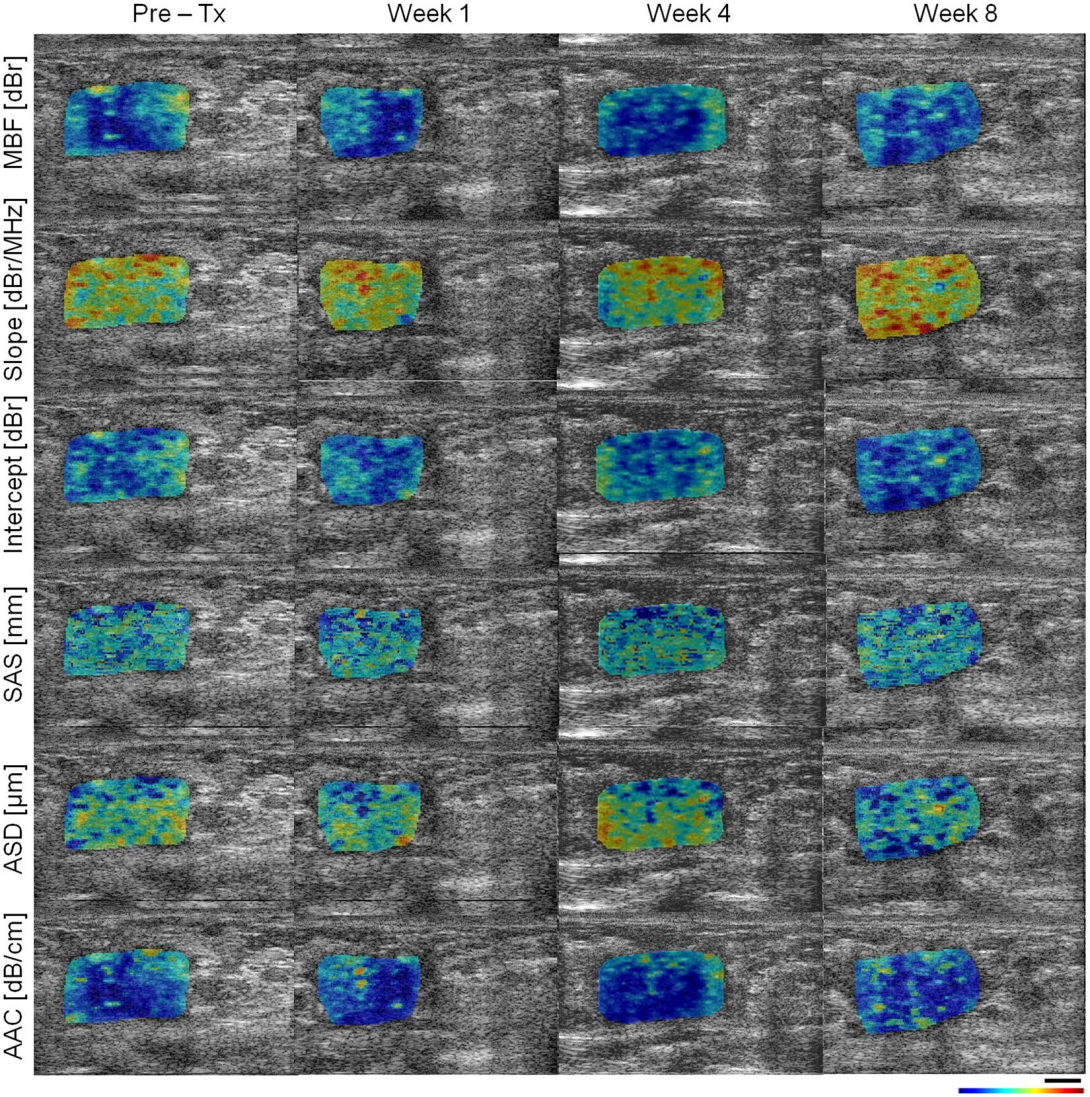
Representative ultrasound B-mode images with QUS parametric overlays (described in Fig. 1) acquired from a non-responding patient prior to and at different times after the start of chemotherapy. The color bar represents a scale encompassing 35 dBr for MBF, 10 dBr/MHz for slope, 65 dBr for intercept, 3 mm for SAS, 150 µm for ASD, and 55 dB/cm^3^ for AAC. The scale bar represents 5 mm.

Figures 1 and 2 demonstrate representative ultrasound B-mode images with QUS parametric overlays of MBF, slope, intercept, SAS, AAC and ASD, obtained prior to and at weeks 1, 4 and 8 after the start of chemotherapy from responding and non-responding patients, respectively. An overall increase in tumour echogenicity was detectable in responding patients after chemotherapy, as observed in parametric maps of MBF, intercept and AAC. No such increase was observed in the case of non-responding patients. Considerable changes were also observed in the textural characteristics of the QUS parametric maps acquired from the responding patient during the course of treatment, compared to pre-treatment (discussed further below).

**Figure 3:**
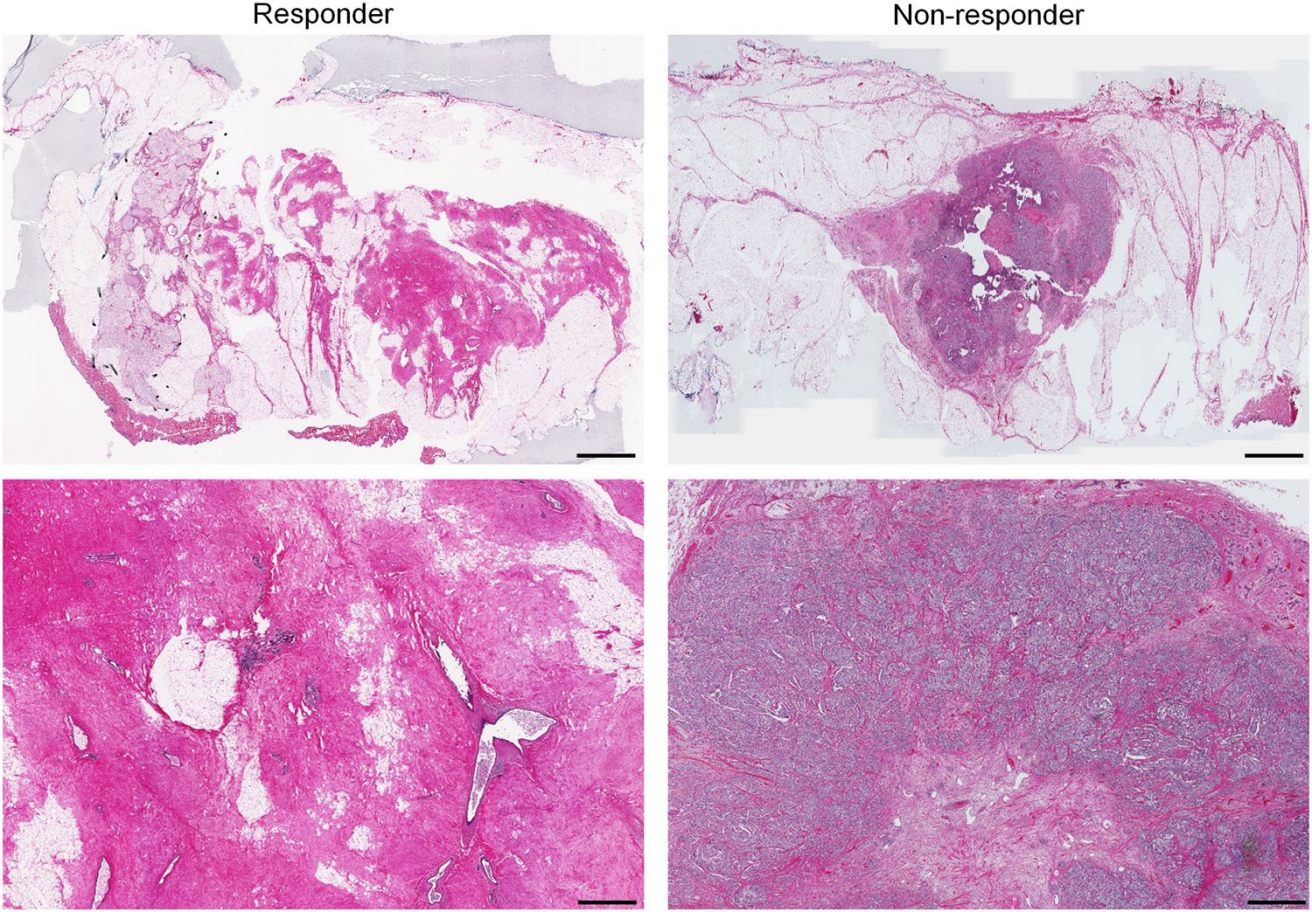
Whole mount histopathology images from representative responding and non-responding patients, at low (top) and high (bottom) magnifications. The responding tumour has been completely degenerated by treatment. A large dense tumour with high cellularity is still present in histopathology images of the non-responding patient. The scale bars represent 5 mm and 500 µm in low and high-magnification images, respectively.

Low- and high-magnification microscopy images acquired from whole-mount histopathology sections of surgical specimens for representative responding and non-responding patients are presented in Fig. 3. In the histopathology images of responding patients, chemotherapy effect was clearly detectable with minimal tumour cellularity remaining within the tumour bed. In contrast, histopathology images of non-responding patients indicated typically large areas of residual disease with minimal chemotherapy effects.

**Figure 4:**
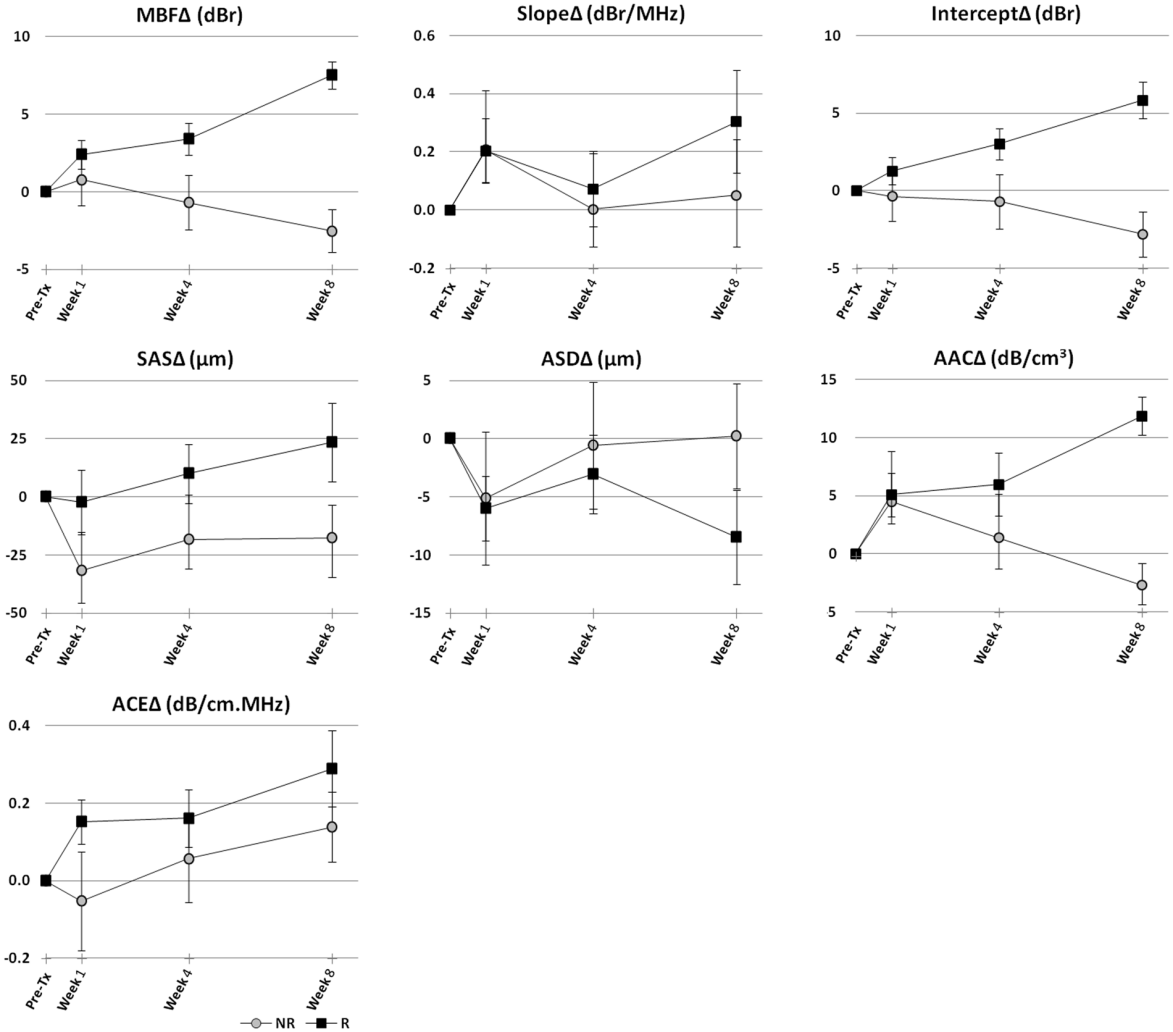
Average data obtained from treatment responding (R) and non-responding (NR) patients, demonstrating changes in QUS mean-value parameters during the course of treatment. Error bars represent ± one standard error of the mean.

**Figure 5:**
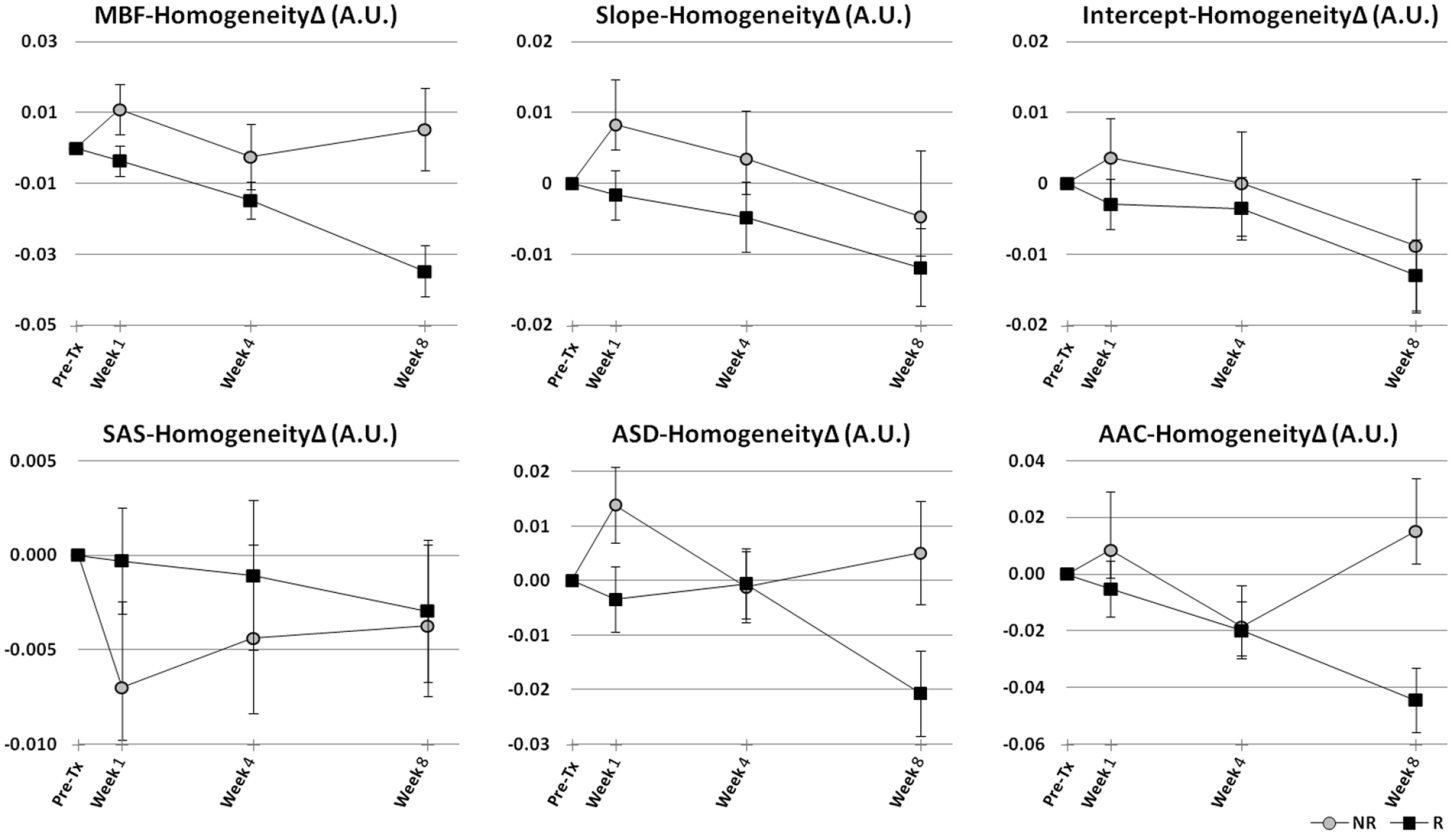
Average data obtained from responding (R) and non-responding (NR) patients, demonstrating changes in homogeneity measure of the QUS parametric maps during the course of treatment. Error bars represent ± one standard error of the mean.

Average QUS data obtained from responding and non-responding patients over the course of treatment is presented in Figs 4 and 5. Figure 4 demonstrates the average changes from the baseline in QUS mean-value parameters for the two patient populations. Among the mean-value parameters, the MBF, intercept, SAS and AAC provided the best separations between the responding and non-responding patients that consistently increased over the course of treatment. The ACE parameter also provided a good separation between the two patient cohorts especially at weeks 1 and 8 of treatment. Table 2 summarizes the average changes in these parameters over the course of treatment for responding versus non-responding patients. Figure 5 demonstrates changes in a QUS textural parameter between the two patient groups. Specifically, the average changes in the homogeneity measure of the QUS parametric maps after the start of chemotherapy are presented. The QUS homogeneity parameters could better separate the two patient populations at week 1, and in some cases at week 4 of treatment, compared to their mean-value counterparts. Similarly, other QUS textural features including correlation, energy and contrast, in many cases, demonstrated a better ability to separate the responding and non-responding patients compared to the corresponding mean-value parameters at earlier time-points after the start of chemotherapy (weeks 1 and 4). Changes in other textural features of the QUS parametric maps over the course of treatment have been provided in Supplementary Figures 1–3.

**Table 2:** The average changes (± one standard error of the mean) in the MBF, Intercept, SAS, AAC and ACE parameters at weeks 1, 4 and 8 of treatment for responding (R) versus non-responding (NR) patients.

Feature	Week 1		Week 4		Week 8	
	R	NR	R	NR	R	NR
MBF∆ (dBr)	2.4 ± 0.9	0.8 ± 1.7	3.4 ± 1.0	−0.7 ± 1.8	7.5 ± 0.9	−2.5 ± 1.4
Intercept∆ (dBr)	1.3 ± 0.9	−0.4 ± 1.6	3.0 ± 1.0	−0.7 ± 1.8	5.8 ± 1.2	−2.8 ± 1.5
SAS∆ (µm)	−2.3 ± 13.9	−31.8 ± 16.7	10.0 ± 12.7	−18.3 ± 19.2	23.5 ± 17.0	−17.6 ± 14.1
AAC∆ (dB/cm^3^)	5.1 ± 1.9	4.5 ± 4.3	6.0 ± 2.7	1.4 ± 3.8	11.9 ± 1.6	−2.7 ± 1.9
ACE∆ (dB/cm.MHz)	0.2 ± 0.1	−0.1 ± 0.1	0.2 ± 0.1	0.1 ± 0.1	0.3 ± 0.1	0.14 ± 0.1

Table 3 presents the result of cross-validated response classification at different times after the start of chemotherapy using optimal subsets of QUS parameters identified through the feature selection process. At week 1 of the treatment a combination of three textural parameters (slope-homogeneity, ASD-homogeneity, and SAS-energy) resulted in an accuracy of 82 ± 0.7% and an area under the ROC curve of 0.75 ± 0.1. A feature set of four textural parameters (slope-contrast, AAC-energy, SAS-correlation, ASD-correlation) classified the responding and non-responding patients at week 4 of the treatment with an accuracy of 86 ± 0.7% and an area under the ROC curve of 0.80 ± 0.1. At week 8 after the start of chemotherapy, one textural and three mean-value parameters (AAC, MBF, MBF-correlation, ACE) in combination could differentiate between the two patient populations with an accuracy of 85 ± 0.9% and an area under the ROC curve of 0.89 ± 0.1.

**Table 3:** Results of cross-validated classification of chemotherapy response at weeks 1–4 after treatment using the optimal QUS features.

Week	Optimal Feature Set	Sen (%)	Spc (%)	Acc (%)	AUC
1	Slope-Homogeneity∆, ASD-Homogeneity∆, SAS-Energy∆	83 ± 1.0	81 ± 1.1	82 ± 0.7	0.75 ± 0.1
4	Slope-Contrast∆, AAC-Energy∆, SAS-Correlation∆, ASD-Correlation∆	90 ± 0.7	83 ± 1.4	86 ± 0.7	0.80 ± 0.1
8	AAC∆, MBF∆, MBF-Correlation∆, ACE∆	97 ± 0.7	72 ± 1.6	85 ± 0.9	0.89 ± 0.1

Reported are average sensitivity (Sen), specificity (Spc), accuracy (Acc) and area under the curve (AUC) ± one standard error of the mean.

**Figure 6:**
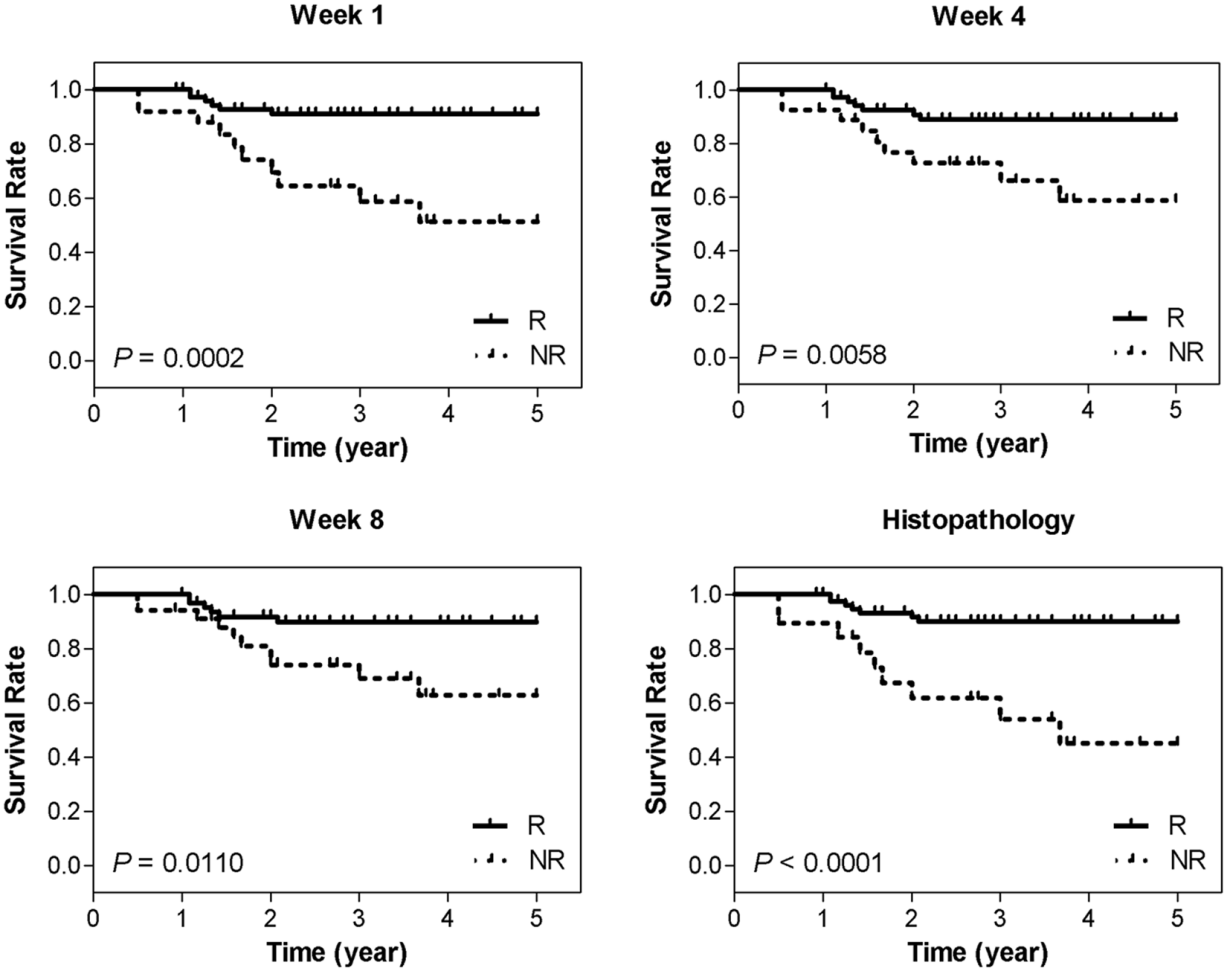
Kaplan-Meier survival curves for responding (R) and non-responding (NR) patients. Patients were classified with cross validation using the optimal QUS feature sets acquired at weeks 1, 4, and 8, and also based on the histopathology at post-treatment.

Figure 6 demonstrates results of recurrence-free survival analyses. The plots present the 5-year survival curves calculated for the responding and non-responding patients classified at weeks 1, 4 and 8 of treatment using the optimal QUS feature sets, compared to those at post-treatment based on the clinical-pathological outcome. A statistically significant difference was observed between the survival curves obtained based on the optimal QUS feature sets at week 1 (p = 0.002), week 4 (p = 0.0058) and week 8 (p = 0.0110) of treatment. Also, a statistically significant difference was indentified between the survival curves obtained months later based on the pathological response of the patients (p < 0.0001).

## Discussion and Conclusions

The results presented in this study from a cohort of 100 patients demonstrated a high accuracy of QUS biomarkers for detecting responses of breast cancer patients to chemotherapy early after the start of treatment. One-hundred women with LABC were monitored during the course of their neoadjuvant chemotherapy and evaluated in terms of standard clinical methods with respect to treatment outcome. The patients were monitored using QUS mean-value and textural parameters as non-invasive surrogates of therapy response over the course of treatment. The pathology-based responses of patients were assessed after surgery using whole-mount sections of surgical specimens. The results demonstrated that pathological responders and non-responders exhibited considerably different trends of change in many QUS biomarkers over the course of treatment. Further, the QUS textural parameters often provided a better separation between the two patient populations early after the start of chemotherapy (weeks 1 and 4), compared to QUS mean-value parameters.

Previous *in vitro* and *in vivo* studies have demonstrated that nuclear condensation and fragmentation in cell death can modify ultrasonic backscatter properties of tissue, at high (above 15 MHz) and at clinically-relevant conventional low (3–9 MHz) frequencies^44, 47, 48, 65^. This is consistent with observations in this study, in which ultrasound backscatter characteristics were quantified by QUS parameters. Banihashmei *et al*. demonstrated that the cellular-based sub-resolution scatterers which alter with cell death can affect ultrasound backscatter signals at low-frequency and summarized the evidence for the role of cell death related nuclear changes^43^.

In the study here, a systematic feature selection process using balanced subsets of data was applied to form optimal QUS feature sets for response classification. These feature sets included three textural parameters for week 1, four textural parameters for week 4 and one textural and three mean-value parameters for week 8 of treatment. Using these small, yet efficient, feature sets, the patient responses were classified with cross-validated accuracies of 82%, 86% and 85% at weeks 1, 4 and 8, respectively. These results imply that the QUS biomarkers investigated in this study have a good potential to be applied for the early prediction of treatment response in patients undergoing cancer-targeting therapies.

The results obtained in this study indicate that changes in the textural characteristics of QUS parametric maps become apparent at early stages after the start of treatment, and will subsequently result in dominant changes in the mean values of these maps. This is in agreement with the observations of our previous study on a small cohort of patients^54^. Early changes in textural properties of QUS images in responding patients may be due to the fact that tumour response to anti-cancer therapies is a gradual process which affects tissue micro-structures heterogeneously. The heterogeneous nature of tumour cell death and response development can influence the textural properties of response-related QUS parametric images, especially at early stages. Previous pre-clinical studies on animal tumour models support the clinical results observed here. In those studies, ultrasound-based textural parameters were found to be more sensitive to cell death, compared to mean values of the spectral parameters^53^. Textural parameters were also capable of detecting changes in tissue micro-structures with a higher correlation to histological cell death, especially at early stages after chemotherapy exposure. A gradual late accumulation of heterogeneous alterations in tissue micro-structures is anticipated to result in more detectable changes in the mean values of the QUS parametric images at later stages^51^. In this study, better separations between the two patient populations were provided by the QUS mean-value parameters at week 8 of treatment, with more mean-value parameters selected within the optimal feature set for response classification.

Ultrasound biomarkers acquired as early as one week after the start of treatment indicated links to patient outcomes in terms of progression-free survival. At weeks 1, 4 and 8, combinations of a few ultrasound-based biomarkers could differentiate between patient outcomes with good agreement with those based on pathology, obtained months later after surgery. Such an early insight into patient outcomes could facilitate the decision of switching to a more effective therapy for treatment-refractory patients or even shifting to a salvage therapy, before it is potentially too late.

In the study here, the breast cancer molecular subtypes were not stratified in response classification based on the QUS biomarkers acquired during the course of chemotherapy. Different molecular subtypes of breast cancer potentially demonstrate various kinetics of response to chemotherapy. Therefore, it would be interesting to investigate the potential impact of molecular subtypes of breast cancer on therapy response evaluation using QUS biomarkers obtained at different times after the start of treatment. A larger cohort of patients with enough number of cases for each subtype is required for such study to permit a valid cross-validated evaluation.

Applications of other functional imaging modalities including MRI, PET, diffuse optical imaging (DOI), and elastography, have been investigated in a number of previous studies for cancer treatment response monitoring^9, 21–24, 66^. Fluorine-18 fluorodeoxyglucose PET (18F-FDG-PET) has been reported to identify response-related changes in tumour as early as 8 days after the start of chemotherapy^67^. In another study, multi-parametric imaging using dynamic contrast enhanced MRI (DCE-MRI) in conjunction with diffusion-weighted MRI (DW-MRI) has been demonstrated a capacity to be used in classifying patient response (n = 33) after one cycle of neoadjuvant chemotherapy with an area under the curve of 0.87^68^. Unlike the most methods based on MRI and PET modalities, the ultrasound-based biomarkers investigated in this study rely on intrinsic contrast alterations arising from changes in the acoustical characteristics of cancer cells when they die, and hence the method does not require injection of any exogenous contrast agents. In addition, ultrasound is a relatively inexpensive, portable and rapid imaging modality that can easily be used to monitor patient response at multiple times during a course of treatment. The QUS techniques proposed here can potentially be implemented as software add-ons on any existing RF-enabled ultrasound system.

The potential of textural analysis techniques to extract relevant features of medical images has recently been reported in other clinical studies of tissue characterization^41, 69^. In particular, the observations of this study on the sensitivity of changes in tissue heterogeneities for an early assessment of treatment response is supported by other studies investigating textural biomarkers of cancer therapy response using MRI^66, 70, 71^, PET^72^, and CT^73, 74^. These studies confirm in principle that early changes in tissue micro-structure and function could be quantified using textural analysis techniques and, consequently, correlated to patient responses to anti-cancer therapies. A similar study has recently investigated the efficacy of textural parameters extracted from diffuse optical spectroscopic (DOS) images to evaluate treatment response in breast cancer patients^24^. Whereas the DOS mean-value parameters have previously indicated the potential to detect response to anti-cancer therapies^22^, textural characteristics of DOS images, taking into account the heterogeneities in tumour physiology and metabolism, have been demonstrated to be more sensitive and specific for the classification of response to treatment. The spatial resolution in DOS imaging (~3 mm) is lower compared to the QUS imaging applied in this study (~200 µm), as the former probes the macroscopic metabolic activities at tissue and vascular levels whereas the latter visualizes microscopic functional activities of cell clusters. Nonetheless, DOS textural biomarkers have also been demonstrated capable of detecting treatment-refractory patients early on during a course of chemotherapy. This further highlights the potential of image-based textural features at different resolutions to quantify changes in tissue micro-structure and functional activities in response to treatment. This potential for QUS parameters to serve as surrogates of response can be applied through a multi-modal multi-resolution imaging framework incorporating various complementary measures of response monitoring, and facilitating a more robust prediction of therapy response as early as possible after treatment initiation.

In conclusion, this study confirms that QUS parametric imaging in conjunction with textural analysis techniques can be clinically applied to predict the pathological response of breast cancer patients to chemotherapy early after the start of treatment. Results indicate that treatment-refractory patients demonstrated different trends in measured ultrasound-based biomarkers over the course of treatment, compared to responding patients. The proposed biomarkers were also found to have a good cross-validated sensitivity and specificity to distinguish patients with poor pathological response to therapy as early as one week following the treatment. The promising results presented in this study imply that these QUS biomarkers, as early survival-linked surrogates of response to cancer-targeting therapies, could be applied to facilitate switching an inefficient treatment regimen to a more effective one on an individual patient basis, within few weeks after the therapy initiation^75^.

## Electronic supplementary material

Supplementary Information
