# Supplementary material for: Chemotherapy-Response Monitoring of Breast Cancer Patients Using Quantitative Ultrasound-Based Intra-Tumour Heterogeneities
Source: Sci Rep. 2017 Sep 4;7:10352. doi: 10.1038/s41598-017-09678-0 (PMC5583340; doi:10.1038/s41598-017-09678-0)
Supplement: Supplementary file 1 — Supplementary Information [file 41598_2017_9678_MOESM1_ESM.pdf]

- Supplementary Information

Chemotherapy-Response Monitoring of Breast Cancer Patients Using Quantitative Ultrasound-Based Intra-Tumour Heterogeneities

Ali Sadeghi-Naini[(1,2,3,4)], Lakshmanan Sannachi[(1,2,3)], Hadi Tadayyon[(1,2)], William T. Tran[(3,5)], Elzbieta Slodkowska[(6)], Maureen Trudeau[(7)], Sonal Gandhi[(7)], Kathleen Pritchard[(7)], Michael C. Kolios[(8)], Gregory J. Czarnota[(1,2,3,4)]

(1) Department of Medical Biophysics, University of Toronto, Toronto, ON, Canada

(2) Physical Sciences, Sunnybrook Research Institute, Sunnybrook Health Sciences Centre, Toronto, ON, Canada

(3) Department of Radiation Oncology, Odette Cancer Centre, Sunnybrook Health Sciences Centre, Toronto, ON, Canada

(4) Department of Radiation Oncology, University of Toronto, Toronto, ON, Canada

(5) Centre for Health and Social Care Research, Sheffield Hallam University, Sheffield, UK

(6) Division of Anatomic Pathology, Sunnybrook Health Sciences Centre, Toronto, ON, Canada

(7) Division of Medical Oncology, Sunnybrook Health Sciences Centre, Toronto, ON, Canada

(8) Department of Physics, Ryerson University, Toronto, ON, Canada

Supplementary Figures

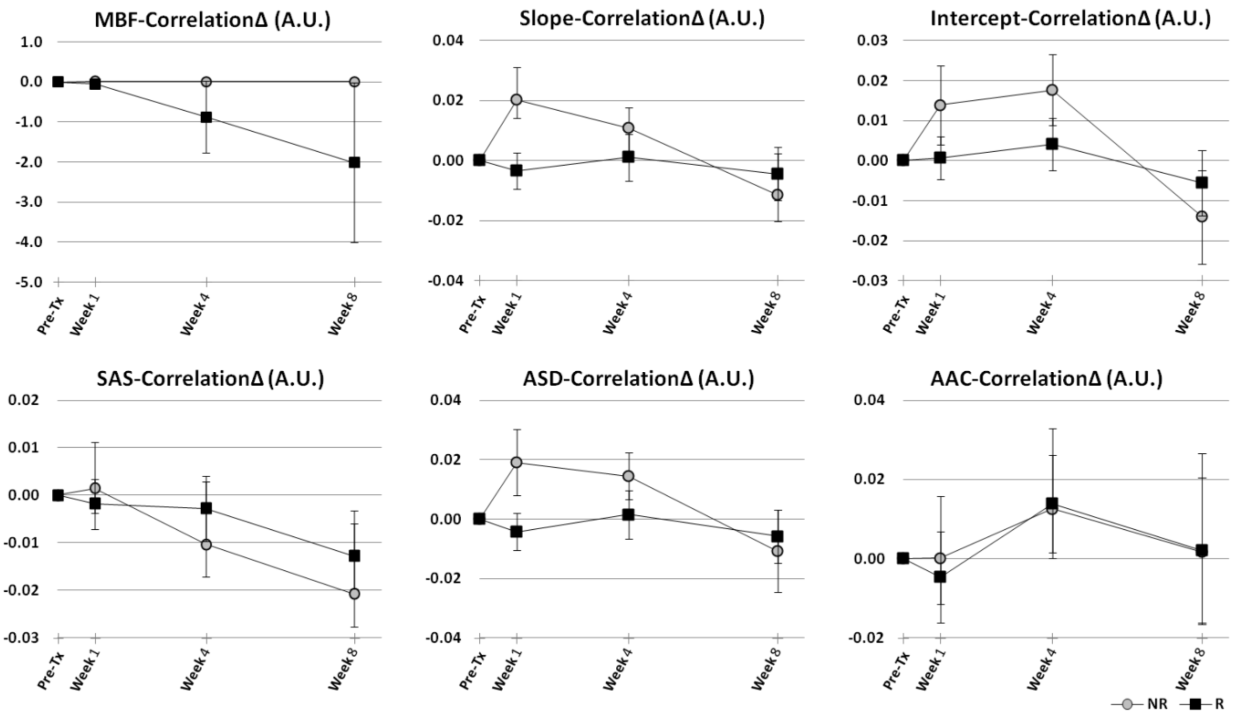

Supplementary Figure 1: Average data obtained from responding (R) and non-responding (NR) patients, demonstrating changes in the correlation feature of the QUS parametric maps during the course of treatment. Error bars represent ± one standard error of the mean.

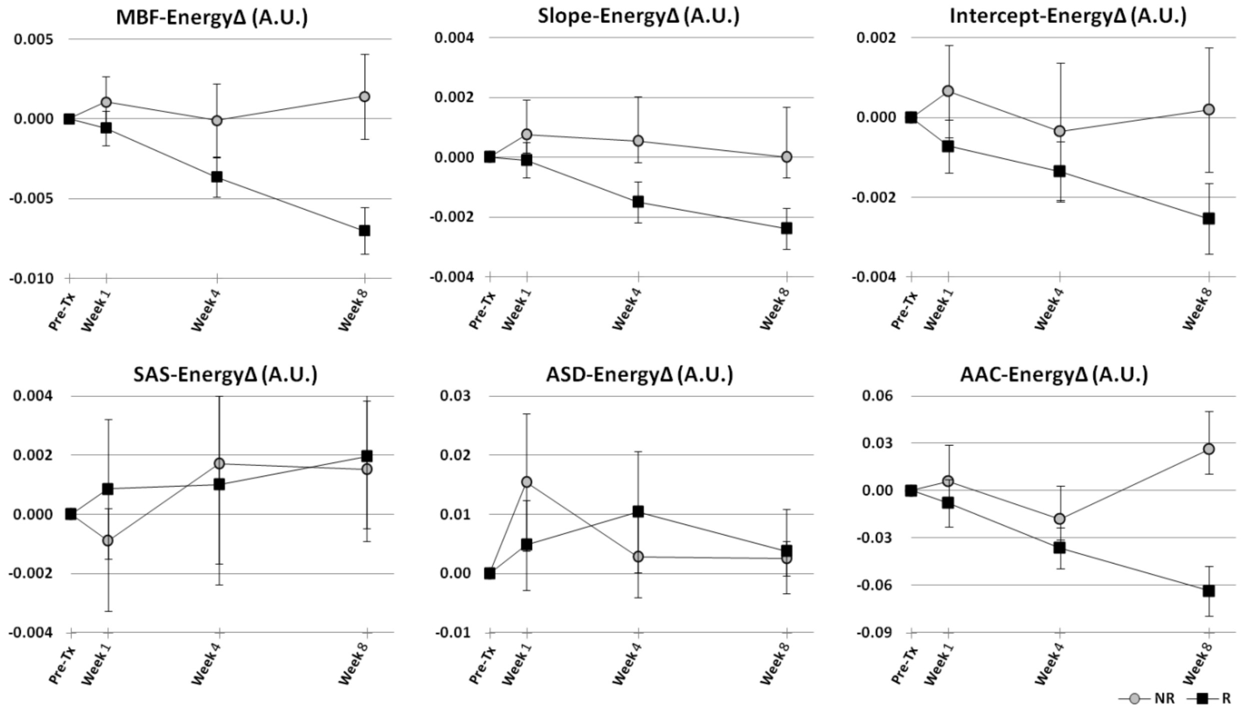

Supplementary Figure 2: Average data obtained from responding (R) and non-responding (NR) patients, demonstrating changes in the energy feature of the QUS parametric maps during the course of treatment. Error bars represent ± one standard error of the mean.

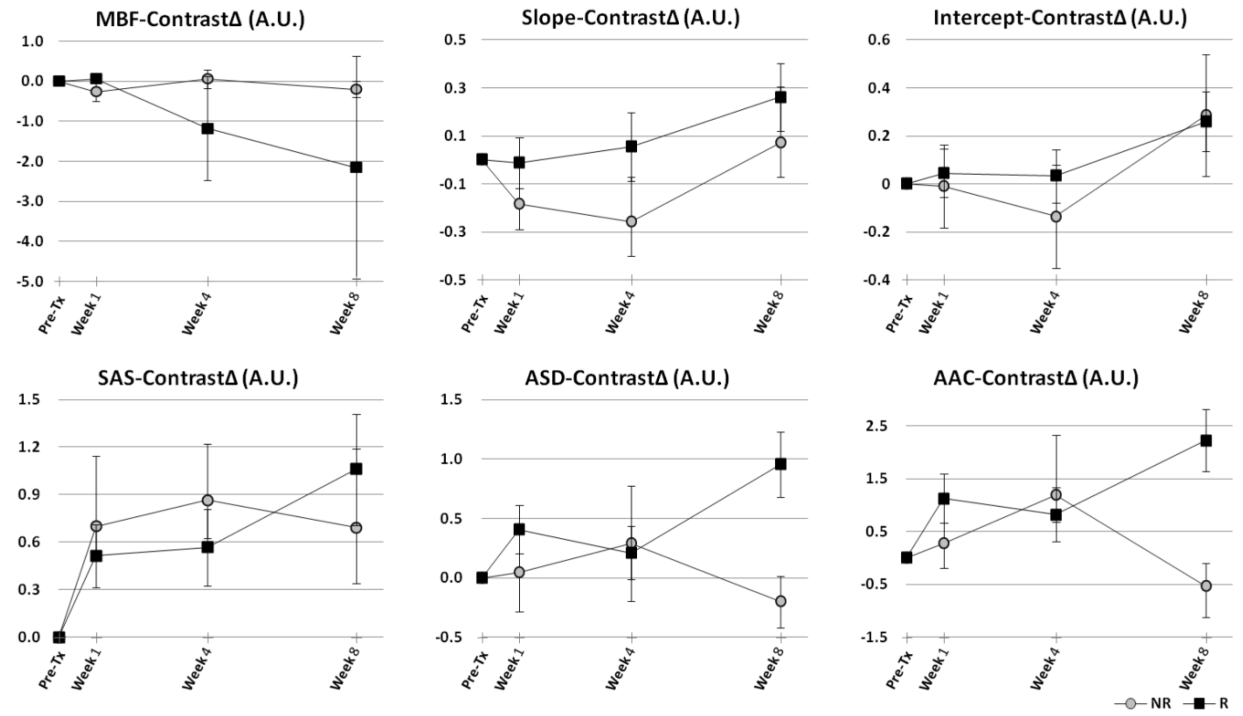

Supplementary Figure 3: Average data obtained from responding (R) and non-responding (NR) patients, demonstrating changes in the contrast feature of the QUS parametric maps during the course of treatment. Error bars represent ± one standard error of the mean.
